# Hydrogel-Based Drug Delivery Nanosystems for the Treatment of Brain Tumors

**DOI:** 10.3390/gels4030062

**Published:** 2018-07-19

**Authors:** João Basso, Ana Miranda, Sandra Nunes, Tânia Cova, João Sousa, Carla Vitorino, Alberto Pais

**Affiliations:** 1 Faculty of Pharmacy, University of Coimbra, Coimbra 3000-354, Portugal; joaobasso@ff.uc.pt (J.B.); ana.alexandramiranda2@gmail.com (A.M.); jjsousa@ff.uc.pt (J.S.); 2 Center for Neurosciences and Cell Biology (CNC), University of Coimbra, Coimbra 3004-504, Portugal; 3 Coimbra Chemistry Centre, Department of Chemistry, University of Coimbra, Coimbra 3004-535, Portugal; snunes@qui.uc.pt (S.N.); tfirmino@qui.uc.pt (T.C.); 4 LAQV REQUIMTE, Group of Pharmaceutical Technology, Porto 4051-401, Portugal

**Keywords:** hydrogel, nanostructured drug delivery system, hydrogel nanoparticles, glioblastoma, drug delivery, local treatment

## Abstract

Chemotherapy is commonly associated with limited effectiveness and unwanted side effects in normal cells and tissues, due to the lack of specificity of therapeutic agents to cancer cells when systemically administered. In brain tumors, the existence of both physiological barriers that protect tumor cells and complex resistance mechanisms to anticancer drugs are additional obstacles that hamper a successful course of chemotherapy, thus resulting in high treatment failure rates. Several potential surrogate therapies have been developed so far. In this context, hydrogel-based systems incorporating nanostructured drug delivery systems (DDS) and hydrogel nanoparticles, also denoted nanogels, have arisen as a more effective and safer strategy than conventional chemotherapeutic regimens. The former, as a local delivery approach, have the ability to confine the release of anticancer drugs near tumor cells over a long period of time, without compromising healthy cells and tissues. Yet, the latter may be systemically administered and provide both loading and targeting properties in their own framework, thus identifying and efficiently killing tumor cells. Overall, this review focuses on the application of hydrogel matrices containing nanostructured DDS and hydrogel nanoparticles as potential and promising strategies for the treatment and diagnosis of glioblastoma and other types of brain cancer. Some aspects pertaining to computational studies are finally addressed.

## 1. Introduction

Glioblastoma (GBM) is the most common, aggressive and lethal type of brain cancer, presenting a highly diffuse infiltrative behavior on neighboring structures [1]. This particular behavior, along with the sophisticated chemotherapeutic drug resistance mechanisms, may be part of the basis of the high recurrence rates after tumor resection surgery, chemotherapy and radiotherapy, with surviving cancer stem cells spreading and colonizing other tissues. The lack of drug variety and efficacy in therapeutic regimens also contribute to the fast progression of this disease, with patients who undergo triple therapy surviving no longer than 15 and 31 months when diagnosed with primary and secondary GBM, respectively [2,3].

GBM current local therapy is limited to the use of carmustine wafers (Gliadel^®^), being implanted in the brain cavity after resection surgery. These polifeprosan 20 (1,3-*bis*-(*p*-carboxyphenoxy)propane and sebacic acid in a 20:80 ratio) implants protect carmustine from degradation and are responsible for a controlled release over 3 weeks of the drug into the brain cavity [4]. Although Gliadel^®^ remains a safer and more effective strategy than intravenous administration of carmustine, local side effects, drug resistance, poor drug penetration in brain tissue, rapid drug release, implant dislodgement, and the invasive nature of the procedure label this system as non-ideal for GBM therapy, hence the need of novel and improved systems [4,5,6].

The intrinsic and adaptable properties of hydrogels have credited these systems as promising and effective strategies against several types of neoplastic diseases, including GBM and other types of brain cancer. Hydrogels are 3D structures formed by crosslinking of hydrophilic polymer chains that become hydrated in aqueous media, thus resembling, to a large extent, a biological tissue [7]. In fact, hydrogels are insoluble in water or other solvents after the establishment of covalent or physical interactions between the polymer chains, which prevent their dissolution. The introduction of more or less labile bonds in the hydrogels leads to the production of a more or less stable system, with service life, gel strength and sustained release properties able to be modulated according to the intended use [8]. In addition, properties such as biocompatibility and biodegradability are regarded as fundamental in hydrogel therapy, considering the long exposure of the gel to cells and biological tissues [9]. Chemical modification of the polymers with stimuli-responsive functional groups is already a frequent approach to functionalize and tailor hydrogels to a wide range of possible applications, such as tissue engineering and wound repairing, cell culture and drug delivery [8,9,10].

It is not surprising that hydrogels, as drug delivery systems (DDS), have been mostly used to carry hydrophilic drugs, given their equally hydrophilic nature [11,12]. Yet, if we consider the pharmacological class of antineoplastic agents, we notice that most of them are poorly soluble in aqueous media [13]. Such constitutes the major limitation of the use of hydrogels as anticancer therapy, due to the incompatibility between their hydrophilic matrices and hydrophobic drugs, which generally results in low encapsulation of these same drugs. However, this problem has not been an obstacle to betting on hydrogels as anticancer therapy in GBM, and many efforts have been made to overthrow this issue [14,15]. 

**Figure 1:**
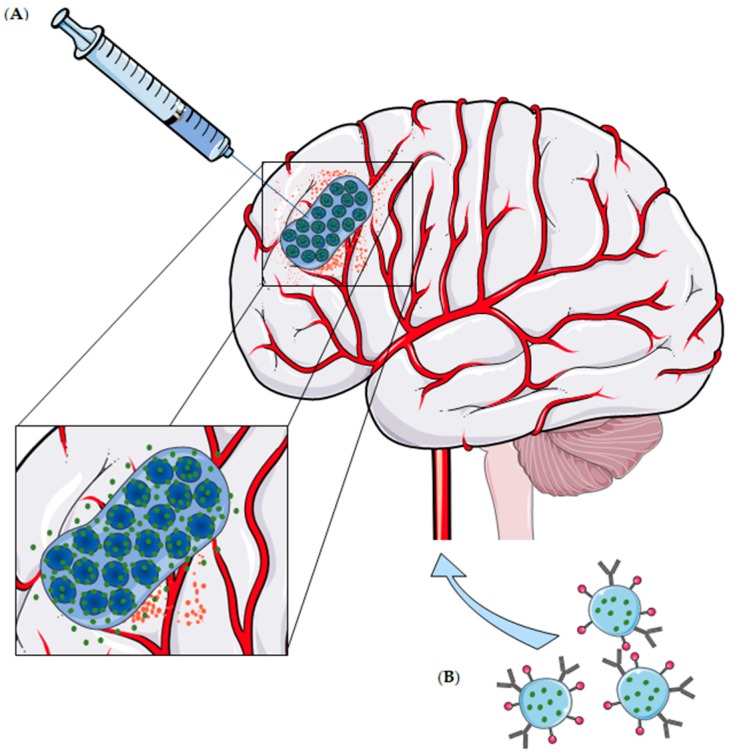
Administration of hydrogels for glioblastoma (GBM) and/or other brain tumors treatment. (**A**) Local delivery of nanostructured systems loaded in a macroscopic hydrogel matrix, via intratumoral injection or implant after surgical resection. Note that a controlled and localized release of the therapeutic agent over time is claimed. (**B**) Intravenous administration of hydrogel nanoparticles with surface functionalization as an active targeting strategy.

Over the last decades, nanocarriers have been intensively studied as a therapeutic approach against brain cancer, including inorganic, lipid and polymeric nanoparticles, micelles, liposomes, dendrimers, nanotubes, among others [16,17]. Nanocarrier size, composition and surface characteristics may be modified, awarding them the ability to cross the blood-brain barrier (BBB) following a systemic administration, hence delivering hydrophilic and/or lipophilic molecules to brain cells. In addition, they provide a sustained drug release and protect drugs against degradation, significantly increasing their half-life time while reducing toxic effects [17]. Interestingly, the combination between nanocarriers and hydrogels is emerging as a novel approach against brain cancer, as these hybrid systems grant a concurrent and controlled delivery of multiple hydrophobic drugs to the tumor, via implantation after surgical resection or by intratumoral injection [11,12,15]. Despite the promising results of these macroscopic systems, more ambitious structures may be designed to overcome the need of invasive procedures during the administration of macroscopic gels. The polymeric nature of hydrogels is, by itself, an opportunity to design independent and nanosized structures to drug delivery. These hydrogel nanoparticles have emerged as a more sophisticated platform, retaining both the hydrogel characteristics and the advantages of nanoparticles, including the feasibility of intravenous administrations and a tight control over drug delivery [18] (Figure 1).

Hydrogels have also been used as 3D cell models for GBM, as a tool to rebuild the tumor architecture in vitro. These systems are a low cost, but reliable alternative to improve the understanding of disease behavior and the communication and interactions in the tumor microenvironment [19,20,21]. In addition, they have proven to be suitable for anticancer drug screening testing, being a potential tool for preclinical assessment of new therapies [22].

The following subsections describe the current state-of-the-art applications of these hydrogel-based systems in GBM therapy (Table 1). PRISMA guidelines [23] were followed to systematize the information used in the present review, in which only hydrogels containing nanostructured DDS or nanogels have been taken into consideration. However, there are some higher-scaled arrangements described in literature with proved application against GBM, particularly, with the use of microspheres [24,25,26,27].

## 2. Hydrogel Matrices Containing Nanostructured DDS

Injectable hydrogels have been considered for the past decades as DDS, due to their aforementioned characteristics. Based on their gelation mechanism, they may be classified into physical or chemical hydrogels [51,52]. The presence of covalent bonds in chemical hydrogels leads to a higher mechanical strength and stability, which is associated with a poor response to medium stimuli [53,54]. Consequently, physical hydrogels, crosslinked by non-covalent interactions, have been extremely attractive for cancer therapy, including against GBM. The majority is stimuli-responsive, being subject to transitional changes in response to environmental stimuli (whether temperature, pH or ionic strength), thus controlling the release of therapeutic agents [15,55,56]. In the case of physical thermosensitive hydrogels, they are intended to present a sol-gel transition temperature close to the body temperature [12]. An interesting example is OncoGel™, a thermosensitive gel depot system containing paclitaxel (PTX), that was tested in rats with intracranial gliosarcoma, resulting in an increased number of long term survivors, when given with temozolomide (TMZ) and/or radiotherapy [15,57]. In addition to the hydrophilic polymers, such hydrogels generally also contain hydrophobic domains being able to incorporate simultaneously drugs with different solubilities. While the known poly(ethylene glycol) (PEG) provides water solubility to hydrogels, other polymers like as poly(lactic acid) (PLA), poly(ε-caprolactone) (PCL), poly(propylene oxide) (PPO), poly(d,l-lactide-*co*-glycolide) (PLGA) and poly(ε-caprolactone-*co*-d,l-lactide) (PCLA) are commonly used to assign hydrophobicity [11,12,56].

Despite drugs can be directly incorporated into the hydrogel, drug-loaded nanoparticles have been increasingly combined with macroscopic hydrogels to form a hybrid system, aiming to achieve a longer sustained drug release [10,58]. The following subsections describe nanostructured systems, which have been loaded in hydrogel matrices as a therapeutic approach against brain tumors.

### 2.1. Polymeric Micelles

As it is well-known, polymeric micelles are formed upon self-assembly of amphiphilic copolymers, giving rise to nanostructures with a hydrophilic exterior and hydrophobic core. They are attractive by the ability to encapsulate poorly soluble drugs in the core, reason why they have been widely used as DDS in various therapeutic areas, including cancer [59,60].

A photopolymerizable poly(ethylene glycol) dimethacrylate (PEG-DMA)-based hydrogel was designed as a strategy to locally deliver TMZ into the brain. The first step considered the preparation of TMZ-loaded PEG_750_-(Poly(ε-caprolactone-*co*-trimethylene carbonate)) polymeric micelles (M-TMZ), aiming to promote a better solubilization of the hydrophobic drug, followed by the formation of M-TMZ-loaded hydrogels (M-TMZ/PEG-DMA). Such system was designed to be injected and quickly photopolymerized in GBM resection cavity, resorting to the use of UV light. Here, TMZ release profile was found to be similar to the reference Gliadel^®^, with TMZ experiencing a burst release of 45% during the first 24 h, and a logarithmic release of 20% over the first week. In addition, such system exhibited good in vivo antitumor efficacy, marked by high apoptosis and loss of tumor mass in xenograft U87 MG tumor-bearing mice [28].

The design of a macroporous hydrogel, containing PTX loaded in methoxyPEG-poly(d,l-lactide) micelles is a strategy planned to improve the delivery of PTX and, at the same time, to inhibit GBM recurrence. Micelles were produced through an emulsion/solvent evaporation technique, to which was further added gelatin to form the hydrogel. In vitro release studies comparing free micelles and micelles loaded in the hydrogel indicated a higher sustained release provided by the hydrogel system, with a reduced burst effect. In another in vitro test, the hydrogel was found to be completely degradable in the presence of collagenase, thus also favoring the release of PTX. Studies in C6 cell line confirmed the applicability of the hydrogel system. Micelle-loaded hydrogel showed to be more toxic to cancer cells than PTX alone, probably due to an increased cellular uptake of micelles and a rapid efflux of free PTX from tumor cells. Altogether, this hydrogel nanocomposite is a potential system to improve the therapeutic efficacy of PTX in the resection cavity following surgery [29].

### 2.2. Polymeric Nanoparticles

Another type of polymer-based nanomedicine are nanoparticles, in which drugs can be either encapsulated within their polymeric matrix or adsorbed on their surface. There is currently a wide range of biodegradable and biocompatible polymers suitable for the preparation of such systems, along with different types of ligands that can be conjugated with nanoparticles for targeted therapies [61,62].

In an attempt to enhance and target gene delivery by lentiviral vectors, their immobilization in hydroxylapatite (HA) nanoparticles within a collagen hydrogel proved to be a useful strategy. The main goals involved studying virus activity and in vitro cell transduction with both collagen- and HA/collagen-based hydrogel. These hydrogels are expected to be implanted into the tumor area, with the subsequent infiltration of surrounding cells, reason why in vitro studies should simulate such ability of malignant cells to infiltrate and migrate into the gel. Initially, it was verified that collagen gels with a concentration ≥0.15% (*w*/*v*) were able to retain and stabilize lentivirus, while maintaining a high transduction activity in invasive C6 glioma cells (approx. 80% of the control). Based on previous results, HA nanoparticles were thought to bind and retain the virus within the gel more effectively. In fact, this was confirmed when HA was added to the hydrogel, increasing the lentivirus activity for 72 h. Furthermore, the release of lentivirus from hydrogel with and without HA significantly diverged, being faster in the second case. Higher solid content in collagen gel was also associated with a slower release profile [30].

Another curious example relates to the use of a magnetic resonance imaging (MRI) traceable ultra-thermosensitive hydrogel, composed of negative charged carboxymethyl cellulose (CMC)-grafted poly(*N*-isopropylacrylamideco-methacrylic acid) (CMC-g-PNIPAAmMA) and positive charged gadopentetic acid/branched polyethylenimine (DTPAGd/bPEI) incorporating epirubicin as hydrophilic drug (hydrogelGd/EPI), that simultaneously integrates bovine serum albumin (BSA) nanoparticles encapsulating paclitaxel, as hydrophobic drug (BSA/PTX). This system is undoubtedly innovative given the unique features that allow it to be used for in situ drug delivery or in the residual tumor tissues after surgical resection, aiming to prevent recurrence of the disease. The most important one, its lower critical solution temperature (LCST) value ranging between 26 and 28 °C, favors the rapid gelation of the hydrogel in tissues (contrarily to common thermosensitive hydrogels that have LCST ≥ 32 °C, and gel-forming in tissues is often compromised due to the conditions in the operating room). Further, thanks to the use of gadopentetic acid and its magnetic resonance contrast ability, the distribution in tumor tissues and degradation of hydrogel can be monitored in real-time. Although the use of poly(*N*-isopropylacrylamide) (PNIPAAm) scaffolds may induce cytotoxicity in vitro, thus suggesting a toxic reaction following implantation in vivo and biocompatibility concerns [63], the hydrogel itself, containing a methacrylic derivative of PNIPAAm was found to be non-toxic and without hemolytic activity. Moreover, the hydrogel itself was found to be non-toxic and without hemolytic activity. When tested in human brain tumor MBR 614 cells, it proved to be a successful dual-DDS releasing in a sustained manner both EPI and PTX, stage-by-stage, with the subsequent inhibition of tumor cells growth. Such fact was confirmed by IC_50_ values, being 1.62, 19.94, 13.46 and 0.85 μg/mL for free EPI, hydrogelGd/EPI in 24 h, BSA/PTX nanoparticles incorporated hydrogelGd/EPI in 24 h and 48 h, respectively. In vivo studies were performed with two different models (MBR 614 tumor-bearing and human glioma U87 MG tumor-bearing mice) and showed that the implantation of the BSA/PTX nanoparticles incorporated in hydrogelGd/EPI resulted in a remarkable improvement of the average survival, as wells as an effective tumor reduction and recurrence prevention, when compared to control group, free EPI and unloaded BSA NPs incorporated in the hydrogel [31].

**Figure 2:**
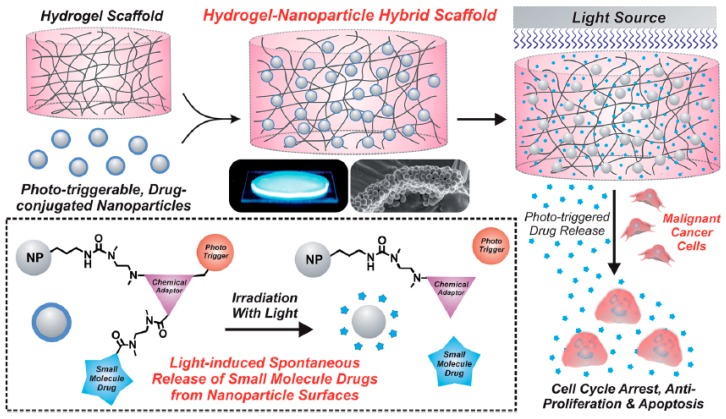
Schematic illustration depicting a poly(ethylene glycol) (PEG) based-hydrogel matrix loaded with silica nanoparticles. Upon photo-irradiation of the hydrogel, covalently bounded camptothecin (CPT) is effectively released, thus inducing U78 MG cell death. Retrieved from ref. [32] with permission from Royal Society of Chemistry.

Shah S. et al. [32] developed an interesting photo-triggerable hybrid platform (silica nanoparticles encapsulated in PEG-based hydrogel) for camptothecin (CPT) release, which was later tested in human U87 MG cells expressing a mutant epidermal growth factor receptor vIII (EGFRvIII). A photo-triggerable chemical adaptor was synthetized using 4-hydroxymandelic acid and then covalently bounded to CPT. This complex was gated to the silica nanoparticles surface, and subsequently encapsulated within the hydrogel matrix. Upon photo-irradiation, it was expected an activation of the chemical adaptor, capable of breaking the covalently-bound drug and allowing its release from the PEG-based hydrogel. This phenomenon was confirmed with a GBM cell line, where there was a marked decrease in viability in the cells exposed to UV light, compared to those non-exposed (see Figure 2) [32].

Pluronic^®^ F127 (PF127) is a commercial poloxamer comprising units of ethylene oxide (PEO) and propylene oxide (PPO), whose biopharmaceutical application is essentially due to its known thermosensitivity. As such, PF127 is an aqueous solution at room temperature that immediately changes to a semi-solid, rigid gel state after parenteral administration, when it comes into contact with body temperature [33,64]. Xu Y. et al. [33] designed PEG-PLGA nanoparticles combined with a PF127-based hydrogel to co-deliver PTX and TMZ. Bearing in mind the solubilities of both drugs, they were incorporated simultaneously in the nanoparticles resorting to a double emulsification/solvent evaporation method. Moreover, compounds such as Pluronic^®^ F68, sodium alginate and hydroxypropylmethylcellulose were added to PF127 solutions to adjust gelation and rheological properties. The release of both drugs was found to be dependent and controlled by the composite hydrogel corrosion. Regarding in vitro studies with U87 MG and C6 cell lines, it was concluded that the gel promoted the most potent growth inhibiting and apoptosis-inducing effects. For PTX/TMZ solution, PTX/TMZ-nanoparticles and the gel, the apoptosis rates in U87 MG cells were 23.6%, 26.4% and 32.5%, whereas in the C6 cells were 26.0%, 30.0% and 39.2%, respectively [33].

### 2.3. Magnetic Nanoparticles

Magnetic nanoparticles have shown potential as personalized therapeutic approaches for different biomedical applications, including the area of cancer. Possessing small particle size and magnetic properties, such particles can be modulated to react to certain magnetic field gradients, generating a desired effect in the body. Magnetic nanoparticles can be used for different purposes, from diagnosis (imaging) to therapeutic (drug delivery, magnetic hyperthermia, photodynamic and photothermal therapy) fields, which makes them extremely appealing systems [65,66,67]. Among the most explored, are the iron-based nanoparticles, specifically magnetite (Fe_3_O_4_) and maghemite (γ-Fe_2_O_3_), due to better biocompatibility and biodegradability profiles [66,68].

**Figure 3:**
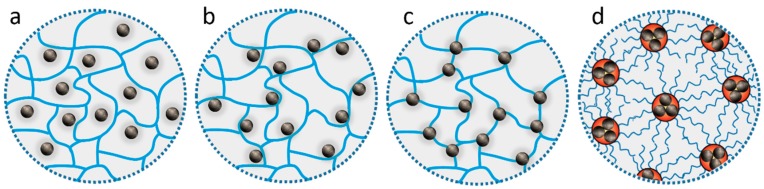
Schematic illustration of polymer gel frameworks containing nanoparticles, including a chemically crosslinked network with (**a**) entrapped particles and (**b**) the physical interactions between magnetic particles and polymer, (**c**) a hybrid network with particle crosslinkers, and (**d**) micelles loading magnetic particles.

Figure 3 illustrates the methods used for the incorporation of magnetic particles into a gel, forming different magnetic gel architectures. These include (i) particles entrapped by the polymer meshes, displaying weak interactions, (ii) chemically/physically crosslinked polymer with entrapped particles, showing stronger interactions (e.g., hydrogen bonds), particles crosslinked in the polymer network by covalent bonds, and (iv) particles loaded into micelles, forming an ordered structure by self-assembly.

Meenach S.A. et al. [34,35] developed a stealth system constituted by magnetite nanoparticles entrapped within a PEG-based hydrogel, for dual PTX delivery and hyperthermia, after taking into consideration the clinical advantages of increasing tumor temperature (41 to 45 °C). First, hydrogels were prepared through the polymerization of various units of PEG methyl ether methacrylate (MMA) cross-linked with units of PEG dimethacrylate (DMA) in a dispersion containing Fe_3_O_4_ nanoparticles. Then, hydrogel nanocomposites were added to a solution containing PTX. Due to their ability to heat when exposed to an alternating magnetic field, they can be useful in remote-controlled heating by generating hyperthermia around the tumor. As expected, the crosslinking of hydrogel networks influenced both heating and PTX release: systems with higher crosslinking density were associated to a lower swelling ratio, along with a greater heating extent and a slower PTX release. No synergistic or addictive cytotoxic effects were achieved with a combined therapy in M059K glioma cells, being the results similar to PTX alone. Yet, these Fe_3_O_4_ nanoparticles entrapped within the hydrogel matrices proved to be effective in delivering PTX simultaneously with hyperthermia therapy [34,35].

Further, a MRI-monitored long-term therapeutic hydrogel (MLTH) system was created by Kim J.I. et al. [36,37], which was no more than a combination of a thermosensitive/magnetic poly(organophosphazene) (PPZ) hydrogel loaded with PEGylated cobalt ferrite (P-CoFe_2_O_4_) nanoparticles, as an imaging platform, and the SN-38 (active metabolite of irinotecan) as chemotherapeutic agent. This hydrogel, containing P-CoFe_2_O_4_ nanoparticles, was produced through hydrophobic interactions, and the final formulation was then obtained via physical mixing of SN-38 with the magnetic hydrogel. Through in vitro release assessment, it was concluded that by monitoring both the polymer concentration and the SN-38 amount, the MLTH platform can release the drug in a sustained manner due to a long-term biodegradation of the hydrogel. When tested in U87 MG tumor-bearing mice over 22 days, the MLTH induced a longstanding inhibition of tumor growth. In short, such system is intended to be injected with a reversible sol-gel phase transition close to the body temperature, promoting a sustained drug release and functioning as MR imaging. The latter property provides a spatial and temporal notion about the MLTH-treated and the MLTH-untreated areas of GBM in MR images over time [36,37].

### 2.4. Lipid-Based Drug Delivery Systems

Lipid-based DDS have attracted increased attention, as they are suitable carriers for the delivery of drugs with poor aqueous solubility. Additionally, they are commonly constituted by generally recognized as safe (GRAS) excipients, with little to no toxicity, when compared to polymeric systems [69]. Different systems have been designed, namely, emulsions, vesicular (including micelles, liposomes and nanocapsules) and particulate systems (including mainly solid lipid micro and nanoparticles, nanostructured lipid carriers and lipid drug conjugates) [70,71]. The incorporation of these structures in hydrogels improves drug bioavailability and may provide a sustained drug release, offering a superior functionality to hydrogels. Despite being a promising concept against GBM, only a few strategies have been developed.

An example relies on the use of a commercially available methoxyPEG-liposome encapsulated doxorubicin (DOX) to produce a thermoreversible hydrogel for the treatment of glioma [27]. Other formulations were also designed, including free and loaded DOX microspheres, as well as a combination between free drug and the carrier systems. The biocompatible hydrogel, composed of the copolymer poly(*N*-isopropylamide-*co*-*n*-butyl methacrylate) [P(NIPAAm-*co*-BMA)] and PEG, presents a unique temperature kinetics [72]. Below LCST, the hydrogel is liquid. However, above that temperature, a sol-gel transition can be observed, meaning that the hydrogel may be injected/inserted into the tumor in a liquid state and gelify in situ, at body temperature. Nonetheless, the LCST may be modified by copolymerizing NIPAAm with other monomers [73,74,75,76], therefore modulating the hydrogel according to the desired purpose. An in vitro controlled release was achieved during 52 days for the hydrogel containing loaded liposomes, indicating a clear advantage over the other formulations (30 days for the hydrogel containing microspheres and 12 days for the formulation with free DOX). These promising results were also verified after a single subcutaneous injection, adjacent to the tumor, in human glioma xenograft models. The formulation with free + DOX loaded liposomes successfully inhibited tumor growth for 38 days vs. 32 days for the corresponding free + DOX loaded microsphere hydrogel, while the DOX free hydrogel formulation was only effective for 12 days [27].

Another example is the innovative hydrogel formed by lipid nanocapsules (LNC) and lauroyl-gemcitabine (GemC_12_), intended to be injected inside the tumor or in the tumor resection cavity. Such hydrogel quickly became appealing by the characteristics it gathered. From the point of view of formulation, it is simple and easy to scale up, with biodegradable and biocompatible components. Besides, it does not require the use of extra agents for gelification or solvents during the preparation. Regarding the drug, gemcitabine (Gem) was found to pass the BBB, in addition to having radio-sensitizing properties, thus favoring combined therapy. This hydrogel was subjected to a proof-of-concept study, which revealed encouraging results. Briefly, the in vitro studies show the hydrogel released Gem in an artificial cerebrospinal fluid, in a sustained and prolonged manner. In U87 MG cell line, the IC_50_ values after 48 h of incubation were 12.06 µM, 0.18 µM and 0.56 µM for Gem, GemC_12_ and GemC_12_-LNC, respectively. When injected in a subcutaneous human GBM tumor model, the GemC_12_-LNC hydrogel was able to significantly reduce or even make the tumor disappear [38]. Another research work considered two distinct antitumor efficacy studies with GemC_12_-LNC and confirmed the following findings: first, the hydrogel formulation significantly improved the median survival of animals after intratumoral administration, in U87 MG human xenograft orthotopic model, compared to controls; second, the perisurgical administration of GemC_12_-LNC within the tumor resection cavity led to a decrease in tumor recurrence formations [39].

Recently, a hydrogel containing solid lipid nanoparticles (SLNs) was developed and the selected formulation optimized as a possible candidate for glioma treatment [40]. Considering the epidermal growth factor receptor (EGFR) gene amplification and protein overexpression in GBM cells, the cyclophosphamide loaded SLNs were superficially modulated with anti-EGFR antibodies [77,78]. Interestingly, this hydrogel was designed for an intranasal administration, thus adhering to the nasal mucosa and slowly releasing the SLNs. To our knowledge, it is the only nanometric system loaded in a hydrogel matrix designed for an administration distinct from local delivery. Moreover, this painless administration route bypasses the BBB and allows a direct drug administration to the brain [79]. Despite the promising in vitro results, cytotoxicity assays and in vivo studies are still required to prove its efficacy.

## 3. Hydrogel Nanoparticles

Hydrogel nanoparticles (also known as nanogels) have attracted wide attention in nanomedicine especially for pharmaceutical applications, as they have been reported to be useful in different areas, from passively controlled drug release to targeted drug delivery, stimuli-responsive drug delivery and bioimaging [58,80].

Nanogels are 3D crosslinked polymeric networks that combine the advantages of both hydrogels and nanoparticles. The swelling capacity, hydrophilicity, biocompatibility and stimuli- responsiveness characteristic of hydrogels in addition to the nanoscale size, high drug encapsulation efficiency, surface modulation, minimal toxicity and serum stability of nanoparticles have credited nanogels as one of the next generation of DDS [81,82]. Nanogels, due to their small size, are suitable for intravenous injections, effortlessly cross the BBB and are easily internalized by cells, while macroscopic hydrogels are preferably used for in situ administrations [18]. Moreover, they may encapsulate and protect biomolecules against chemical and enzymatic degradation, while allowing their functionality and the free flow of substrates and products [83]. Consequently, nanogels are novel carrier systems, not only for therapeutic, but also for diagnostic and theranostic purposes, with potential application in GBM. The most prominent characteristics of a hydrogel nanoparticle can be found in Table 2.

The intrinsic characteristics of positively charged hydrogel nanoparticles may promote a spontaneous interaction with the negatively charged moieties of RNA molecules, thus forming nano-polyplexes with good loading capacity and size properties [85]. Moreover, the protection from degradation provided by nanogels and the possible surface modulation strategies make these systems a hopeful approach against several types of cancer, including GBM. Recently, various pH and redox-responsive nanogel formulations have successfully encapsulated miR-34a molecules, which target c-Met, Notch-1/2 and cyclin-dependent kinase 6 (CDK6) genes, commonly overexpressed in GBM [86,87,88]. In vitro, these polyglycerol-based nanoparticles, crosslinked through disulfide bonds and with an average size below 170 nm, promoted the upregulation of miR-34 and downregulation of the targeted genes. Later, in vivo studies conducted in xenograft mice models of GBM supported this information, with the most promising formulation showing a reduction in tumor size growth on day 20 (379 ± 175 mm^3^ vs. 1197 ± 359 mm^3^ in the control group) [41]. However, the study was conducted with an intratumoral administration of nano-polyplexes in abdominal GBM tumor induced models, and systemic distribution of the nanogel, as well as the BBB protective effect were not evaluated.

**Figure 4:**
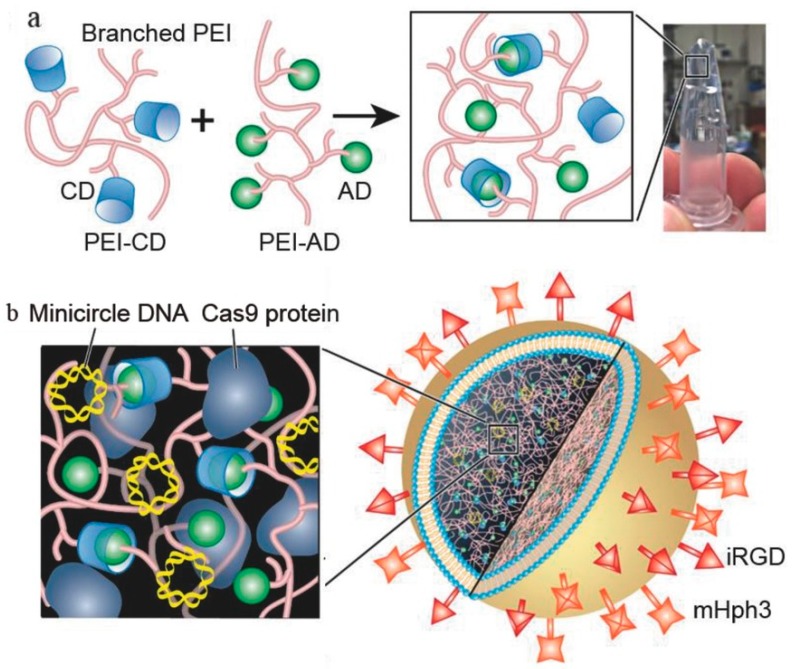
Schematic representation of the production of the liposome-templated hydrogel nanoparticles. (**a**) Cross-linking mediated by cyclodextrin-amantadine host-guess interaction of the polyethylenimine (PEI) chains. (**b**) Hydrogel nanoparticle structure contemplating a hydrogel core encapsulated by a 1,2-dioleoyl-3-trimethylammonium-propane chloride salt (DOTAP) shell. Retrieved from ref. [42] with permission from Wiley Online Library.

Another example relates to a successful tumor delivery of the CRISPR/Cas9 system (which combines a nuclease and a single guide RNA strand) using liposome-templated hydrogel nanoparticles (Figure 4) [42]. A polyethylenimine (PEI)-based hydrogel was crosslinked by host-guest interaction, using cyclodextrin and adamantine-engrafted PEI branches to efficiently increase protein and nucleic acids co-encapsulation by cationic 1,2-dioleoyl-3-trimethylammonium-propane chloride salt (DOTAP) liposomes. These surface modified hydrogel nanoparticles presented a controlled release of DNA and protein over 3 days and proved to be cytotoxic over U87 MG cells. Moreover, intravenous administration of the nanoparticles in orthotopic tumor-bearing mice effectively reduced tumor growth and improved overall survival of the models.

Although some PEI polymers have proved to be suitable carriers for drug and genetic material intracellular delivery, due to their cationic charge, they have also shown some toxicity limitations [89,90,91]. Polyacrylamide-based hydrogel nanoparticles were assembled, with the incorporation of PEI, in order to increase cisplatin (CIS) uptake, while maintaining a lower cytotoxicity promoted by this polymer [43]. In fact, cellular studies performed in 9 L glioma cells demonstrate PEI interacts with cells, resulting in the enhancement of the nanogel internalization, but also in cellular death, in a concentration dependent manner. However, blank nanogels were less cytotoxic than free PEI, by at least a 2-fold factor. Additionally, when loaded with CIS, these particles were more toxic to cancer cells than free CIS, thus being a potential therapeutic option against GBM.

During tumor resection, the identification of neoplastic cells is critical to ensure the best outcome for the patient under surgery. Hydrogel nanoparticles, due to their small size and tumor infiltration potentiality, may be an effective approach to actively target and identify GBM cells. Aiming at tumor visualization, Jiang L. et al. [44] developed a system of Fe_3_O_4_ nanoparticles loaded poly(*N*-isopropylacrylamide-*co*-acrylic acid) (MPNA) nanogels. Its great potential is due to the pH/temperature sensitivity inherent to the magnetic nanogel, whereas a further conjugation with Cyanine5.5 NHS (Cy5.5)-labeled lactoferrin (Lf) introduces a targeted contrast agent for preoperative MRI and intraoperative fluorescence imaging of the tumor. In vitro studies were performed with two different cell lines: C6 glioma cells, with high expression of low density lipoprotein receptor-related protein 1 (LRP1), a known receptor of Lf; and ECV 304 cells with no LRP1 expression. Not surprisingly, both Cy5.5-Lf-MPNA nanogels and Cy5.5-Lf-Fe_3_O_4_ nanoparticles were highly internalized by C6 cell, when compared to MPNA nanogels and Fe_3_O_4_, respectively. The same trend did not occur with ECV 304 cells, as expected, as there were no marked differences among all formulations. One curious aspect is that on both C6 cells and ECV 304 cells, the cellular uptake at pH 6.8 was higher than that at pH 7.4. This can be explained by the fact that Cy5.5-Lf-MPNA nanogels are hydrophilic and enlarged at pH 7.4 (which effectively increases their blood circulation time when compared to Cy5.5-Lf-Fe_3_O_4_), but they convert into a hydrophobic state with lower size at pH 6.8 (tumor microenvironment), thus favoring their internalization by GBM cells. In vivo studies with rats bearing C6 glioma tumors confirmed the previous information, since Cy5.5-Lf-MPNA nanogels revealed an increasing fluorescence signal proportional to the iron concentration. Altogether, it was demonstrated that Cy5.5-Lf-MPNA nanogels can not only target GBM either by passive or active strategies, but also identify and outline the margins of GBM tumors due to their proved MR/fluorescence imaging abilities [44].

Another successful strategy may be the use of Coomassie Brilliant Blue G-250 (CB), a visible contrast enhancer, in polyacrylamide-based nanoparticles, crosslinked with glycerol dimethacrylate. Three different systems were designed following different production methods, namely, CB covalently linked, CB-encapsulated and CB-post loaded nanoparticles [45]. A posterior functionalization with F3 peptides and PEG units was conducted, in order to target GBM cells, due to the high expression of nucleolin in these cells [92,93]. None of the three formulations showed a significant reduction in cell viability. CB covalently linked nanoparticles were considered the best system, due to the highest loading efficiency and lowest dye leaching effect. Furthermore, in vivo studies using rats with brain implanted 9 L cancer cells, supported the efficacy of the optimized system, as a persistent visual tumor delineation was observed until 2 h after the intravenous administration.

Polyacrylamide may also be copolymerized with 2-carboxyethyl acrylate to structure nanogels, with a medium size far below 100 nm, thus being susceptible to be used against brain tumors [46]. In addition, surface functionalization of the particles with the nucleolin-targeting F3 peptide increased their uptake by glioma 9 L cells. In vitro studies conducted with these polyacrylamide-based nanoparticles indicate 42% of DOX was released in the first 24 h, thus exhibiting controlling properties over the delivery of the drug. However, cytotoxicity and in vivo studies are still required to testify the promising results previously described.

Attending to the overexpression of membrane protein connexin 43 (Cx43) and brain-specific anion transporter 1 (BSAT1) of glioma and peritumoral cells, a novel CIS-loaded nanogel, with surface modulation, was developed. Hydrogel nanoparticles were synthesized using PEG-*b*-poly(methacrylic acid) and MAL-PEG-NH_2_ as starting materials, and later conjugated with the antibodies and CIS. Unloaded nanogels were further characterized and showed to have a diameter of 120–130 nm, a polydispersity index of 0.13 and a zeta potential of −15 ± 5 mV, making them suitable for CIS loading, with a loading capacity of 30–35% and an entrapment efficiency of 45%. Additionally, these particles presented a sustained release over time, with approx. 50% of CIS being released after one week. Cellular studies indicated these nanogels have lower toxicity over C6 cells, when compared to free CIS. However, in vivo results showed an increase of overall survival of rats implanted with glioma 101/8 cells and treated with these nanoparticles. It is expected that the antibody-receptor interactions, not only targeted the hydrogel nanoparticles, but also promoted the maintenance of high levels of CIS near the tumor [47].

Poly(vinyl alcohol) (PVA)-based nanogels have failed to meet ideal results due to an inhomogeneous interior, high porosity and lack of polarity. Consequently, divergencies in encapsulation efficiencies and control over release are main drawbacks in the use of these hydrogel nanoparticles [48]. Chemical optimization of molecules and particle surface modification for active targeting are becoming standard requirements for an efficient anticancer activity. For instance, DOX-loaded nanogels assembled by inverse nanoprecipitation after carboxyl-alkynyl and azido-functionalization of PVA, modulated with cyclo(Arg-Gly-Asp) cRGD peptide, showed pH-sensitive and targeting properties for the overexpressed αvβ3 integrin receptors of angiogenic endothelial and GBM cells. Furthermore, these particles were able to control the release of DOX over time, being triggered by low pH and reduction environments, commonly found in GBM [94,95,96]. In vivo results in subcutaneously induced U87 MG tumor bearing mice demonstrated that tumor growth was successfully inhibited by treatment with these hydrogel nanoparticles, while unmodulated nanogels and free DOX failed to prevent this outcome. Moreover, the lower prevalence of adverse effects is a positive mark of a potentially new platform against GBM.

Alginate nanogels co-loaded with gold nanoparticles and CIS were recently developed as an attempt to radiosensitize cancer cells for radiotherapy treatments. Not only the nanocomplex showed higher cytotoxicity on U87 MG cells, comparing to free drug, the platform also showed a marked apoptotic effect after X-ray irradiation on the same cell line [49]. Yet, more studies are essential to prove in vivo efficacy of this nanocomplex.

The use of ferric iron (Fe^3+^) as a cross-linker between the amphiphilic chains of branched pentaerythritol poly-(caprolactone)-*b*-poly(acrylic acid) is an effective strategy to produce hydrogel nanoparticles to effectively delivery DOX to cancer cells [50]. Interestingly, the reduction of ferric iron to ferrous iron (Fe^2+^) in the presence of lactic acid and light (405 nm, 300 mW/cm^2^) compromise the structure of these nanoparticles, thus assigning a light-responsive and on-demand immediate release of the loaded anticancer agent. In vitro tests show these nanoparticles effectively release DOX molecules following laser irradiation, while in the absence of light, the anticancer agent is tightly imprisoned within the particles. Moreover, blank nanogels and loaded nanogels in the absence of light exhibited little toxicity in C6 glioma cells, while irradiated DOX-loaded hydrogel particles effectively decreased cell viability, in a higher extend than free DOX. This may be due to the extensive uptake of these nanostructured systems, when compared to free DOX. C6 tumor-bearing rats were used to confirm the efficacy of the designed nanogels, via tail vein injection. Although the nanoparticles display a wide allocation between the main organs, they were essentially concentrated in the tumor. Moreover, the lack of laser irradiation in healthy organs prevented the release of DOX and therefore, the presence of unwanted effects. Regarding the tumor, the on-demand release of DOX led to an incredible reduction of 91% of the tumor volume, thus assigning these nanogels as a potential and effective therapeutic weapon against GBM.

## 4. Some In Silico Insights

Simulation studies directed specifically to brain tumors are crucial tools that have not been explored so far, and those focusing on the hybrid systems previously described (hydrogel + nanoparticles) are almost inexistent. The major in silico contributions rely on individual hydrogel and nanoparticles characterization and their properties rationalization. In fact, the abovementioned efforts reinforce the need for a deep understanding of recognition, interaction, assembly and transport phenomena in hydrogels and nanostructures, in order to (i) establish the main factors governing the network stability and the adsorption, diffusion and release behaviors, and (ii) to design optimized formulations and therapies directed at GBM. For instance, the delivery of an active compound can be retarded by the occurrence of strong drug-matrix interactions, with a direct effect on the release rate. An accurate estimation of the main interaction components is thus essential for predicting and tailoring such effects. Computational approaches provide adequate reference systems and the most ingredients for accurately controlling the model parameters [97,98,99,100].

In what follows, recent contributions of computational studies and models for understanding relevant aspects on the structural and functional characteristics of nanoparticles and polymer-based hydrogels gels are briefly outlined. The focus will be mainly on the works published in the past three years and the main idea is to highlight the powerful contribution of simulation in the design and optimization of new smart materials towards GBM treatment.

Molecular simulations comprising either coarse-grained descriptions or atomistic models allow decoupling and assessing solvent, drug and polymer specific interactions (e.g., polymer-drug, polymer-polymer, polymer-solvent, and polymer-nanoparticle surface), which are not experimentally accessible. In coarse-graining approaches the polymeric material can be inspected irrespectively of their chemical structure.

The system configurations are defined based on topological data and interaction potentials, in different thermodynamic ensembles, and generated using atomistic or coarse-grained approaches. The latter allow simulating larger systems and use general interaction potentials to describe connectivity, excluded volume and polymer-solvent interactions. For instance, important features previously highlighted, such as the swelling behavior of the hydrogel can be modeled, using a coarse-graining procedures, by inspecting the effect of the polymer chain length, type and density of crosslinking, relative proportion of monomers and the salt effects. On the other hand, atomistic simulations play a major role on the detailed description of solvation and desolvation effects, cosolvency and cononsolvency phenomena, weak noncovalent interactions, hydrophobic effects and hydrogen bonding [101,102].

Monte Carlo simulations (MC) based on the primitive model in which the polymers are represented by bead-spring chains, the ions and chain monomers are represented by hard-spheres and the solvent is considered as a dielectric continuum have been widely used to explore both gel and nanoparticles properties and interactions. Carnal and coworkers [103]) resort to MC to systematically explore the effect of pH, salt valency and NP surface charge density in the interaction between a polypeptide chain with primary structure based on bovine serum albumin and nanoparticles with surface charge densities ranging from −60 to +60 mC/m^2^, aiming at representing systems containing charged inorganic NPs at physiological pH. In the model, each chain monomer represents one aminoacid with a charge dependent on pH and varying from −1 to +1, −1 to +2, or −2 to +1, depending on the number of titrating sites and on the nature of the side-chain. Globally the chain is positively charged at low pH, negatively charged at high pH and neutral at intermediate pH values. It was found that chain adsorption around oppositely charged NPs limits the chain conformational behavior and modifies its acid/base properties. At physiological pH, the complex formation occurs only with positively-charged NPs. For negatively charged NP, the presence of salts, especially those of trivalent cations, screen the attractive interactions between NP and chain, decreasing the complex stability and leading to partially desorbed segments, making the charging process of the chain less efficient. In contrast, for positive NPs, the valency salt effect on the complex stability is almost negligible, due to the absence of competitive effects between multivalent cations and NP.

In a different perspective, Shirakura et al. [104] resort to MC simulations to inspect the relation between the matrix density of hydrogel nanoparticles and the kinetics of drug release, by changing the mesh size through variations in the cross-linking. The study was conducted using CIS as a model drug and the authors found a good agreement between the simulation and the experimental results.

More recently, Stornes et al. [105]) studied the complexation between weak polyelectrolytes and charged NPs to inspect how chain length and concentration and also the polyelectrolyte/nanoparticle ratio influence their interaction. It was found that chain ionization is highly affected by chain length and NP presence. They also found distinct types of NP/chain interactions specially in systems with shorter chains, weak ionization and larger mixing ratios. A competition between chain/NP attraction and intra and interchain repulsion was found. Repulsion between charged monomers promotes either the existence of free chains in the bulk at high pH, in the case of shorter chains or the existence of long tails protruding from the chain−NP complex into the bulk, favoring NP aggregation and precipitation.

In Monte Carlo simulations of gel systems, the polymer network is represented by bead-spring chains connected by covalent or non-covalent bonds between adjacent pairs of particles belonging to different chains or alternatively connected by other entities/smaller chains explicitly introduced to act as linkers. This type of approach has been reported for a long time in this topic and despite its simplicity it remains an actual and useful approach to explore the role excluded-volume and electrostatic interactions in nanogel swelling exploring the influence of gel charge and counterion valence [106], to address the interaction between nanogels, either charged or neutral [107,108] or to establish what governs the inclusion of small molecules in the gel network [109]. In the latter study, it was found, for instance, that steric repulsion dominates for larger cosolutes and for small hydrophobic attractions.

The same type of approach has been used by other authors [110] to the study of the influence of pH on the conformation and ionization of microgels under salt free conditions, varying gel concentration, acidic groups content and crosslinking densities. The degree of ionization, swelling, counterion distribution was evaluated. The authors found that counterion distribution strongly determines the effective charge of the network, thus conditioning the ionization, while the number of acidic groups has only a weak effect on the ionization behavior which in turn influences the degree of swelling.

Recently, our group combined Quality by Design and in silico-in vitro approaches, for developing cationic ultra-small nanostructured lipid carriers (usNLC+) with potential site-specific drug delivery capability towards GBM. The surface modification of usNLC+ was successfully modulated using cationic serine-based surfactants and the respective interaction and internalization behavior, within the BBB, were rationalized by MD and cytotoxicity studies [111]. MD simulations allowed to inspect the effect of cationic surfactants in the stability of lipid membranes and to select the most effective surfactant for interacting and stabilizing the model lipid membrane. From both computational and cell uptake results it was concluded that monomeric surfactants displayed good uptake profiles and that cationic serine-derived surfactants are attractive enhancers of stabilization and transport in the NLCs directed at crossing cell membranes, including those of BBB and GBM cells [111].

The modification of the surface of nanoparticles for targeted drug delivery has been recently reviewed and detailed in ref. [112]. Some computational works have supported the design of responsive nanoparticles, including those decorated with amphiphilic polymers (see e.g., [113,114,115,116,117]). The interaction of designed ankyrin repeat proteins (DARPin) with gold nanoparticles has also been explored by MD simulations using coarse-grained models [118]. This allowed to inspect the adsorption process and the formation of the protein corona. It was shown that the human epidermal growth factor receptor 2 (HER 2) binding domain of the protein was not involved in binding to the nanoparticle. These results in conjunction with the experimental counterpart have suggested that the plasmonic gold nanostructures containing DARPin molecules are attractive nanomaterials for cancer therapy.

Yadav et al. [119] have demonstrated that computational approaches allow enhancing the efficacy of the polymer-based nanoformulations, such as those containing chitosan and cucurmin. This include the selection of the most suitable polymers for loading and encapsulating the therapeutic agent. MD simulations have confirmed the stability of the formulation in aqueous solution. From molecular docking it was concluded that chitosan was the most suitable polymer for curcumin encapsulation with the formation of stable complexes (binding affinity of −4.3 kcal mol^−1^). The modulation of the in-vitro release of the therapeutic agent for exhibiting a sustained release from nanoparticles have direct implications in the prolonged action of drugs against cancer cells.

Other approaches based on dissipative particle molecular simulations have also been employed to describe the formation of self-assembled micelles for anticancer drug delivery, consisting of triblock amphiphilic copolymer methyl poly(ethylene glycol) ether-*b*-poly(β-amino esters)-*b*-poly lactic acid (MPEG-*b*-PBAE-*b*-PLA). Also evaluated were the microstructures at different pH values, and the distribution and release mechanism of DOX [120].

**Figure 5:**
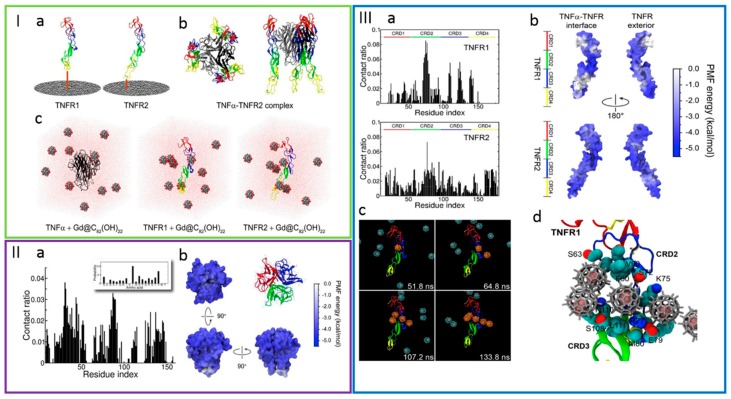
(**I**) Representation of the molecular structures and the simulation systems including (**I**-**a**) TNFR1 and TNFR2 composed of four cysteine-rich domains; (**I**-**b**) the co-crystalized structure between TNFR2 and TNFα; (**I**-**c**) the initial configurations for MD simulations, containing 10 Gd@C_82_(OH)_22_ per protein in water. (**II**-**a**) Site-specific Gd@C_82_(OH)_22_ contacts with TNFα; (**II**-**b**) Potential of mean force (PMF) for Gd@C_82_(OH)_22_ binding to TNFα, projected onto the surface of TNFα. (**III**-**a**) Site-specific contacts with TNFR1 and TNFR2; (**III**-**b**) PMF projection onto the protein surface. (**III**-**c**) Illustration of a representative trajectory corresponding to the binding of Gd@C_82_(OH)_22_ and TNFR1. (**III**-**d**) Illustration of the binding mode for TNFR1. Retrieved from ref. [121] with permission from Elsevier.

The design of cancer therapeutics have also benefited from MD simulations. Zhao et al. [121] have explored the regulation of the tumor necrosis factors signaling pathway by establishing the role of gadolinium-containing fullerenol (Gd@C_82_(OH)_22_) for engaging the cancer cells. MD was employed for rationalizing the leading mechanisms involved in the interaction between tumor necrosis factor (TNFα) and tumor necrosis factor receptors (TNFRs), mediated by a constraining agent for tumor cells (Figure 5). The results suggested that the fullerenol inhibited the TNFα-TNFR1 binding through the association to two of the cysteine-rich domains of the receptor, while the complex formation between TNFα and TNFR2 was enhanced by Gd@C_82_(OH)_22_. The ability of Gd@C_82_(OH)_22_ for controlling the signaling pathway with TNFα-TNFRs at the molecular level was predicted before the experimental observations.

MD simulations have also been employed in the rational design of potential anticancer drugs targeting Glutathione peroxidase-1 (GPx-1) [122]. This included peptide-Au cluster compounds possessing specific peptide sequences gold atoms, which were able to recognize and strongly bind GPx-1. The MD results were confirmed and validated by the synthesis of peptide-Au clusters and by GPx-1 activity suppression studies. The most suitable targeted compound was selected based on the respective affinity to the active site of GPx-1.

The formation and dynamic behavior of different types of hydrogels, under different conditions, has been successfully detailed in silico. For instance, Walter and co-workers [123] have evaluated the effect of the conformation transition of poly(*N*-isopropylacrylamide) hydrogels on the composition of the solvent, in mixtures of water and methanol, suggesting that the cononsolvency was dominated by the strong attachment, via hydrogen bonding, of methanol molecules to the polymer chain. The precipitation of the polymer or the collapse of the hydrogel were thus promoted by the orientation of methanol molecules, i.e., by the hydrophobic effect created by the orientation of methyl groups of methanol toward the bulk solvent [123].

**Figure 6:**
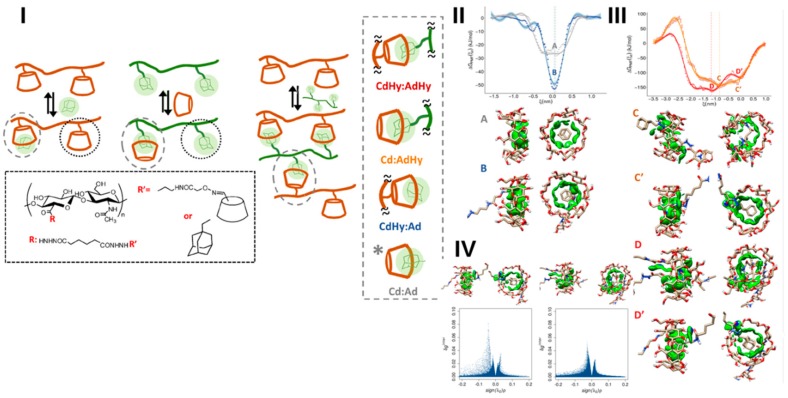
A composed view of the network components and of the inclusion complexes used as junction nodes. (**I**,**II**) PMF profiles for the inclusion complexes between the hyaluronic acid derivatives, (**IV**) 2D scatter plots for the most stable complex, corresponding to the deepest minimum (**left**) and to the higher-lying local minimum (**right**) of the PMF profiles depicted in panel (**III**) (D and D′, respectively). The 3D molecular images for the equilibrium states of the complexes, showing different types and strengths of noncovalent interactions between the system components are also included. Retrieved from ref. [124] with permission of American Chemical Society.

In a different study [124], the modulation of the character of the interaction sites in cyclodextrin-based polymer networks was carried out by incorporating amphiphilic substituents based on hyaluronic acid derivatives (see Figure 6). Our previously designed free-energy oriented method [125], based on MD and potential of mean force (PMF) calculations, was optimized and employed for establishing the underlying thermodynamics signatures and for identifying the stabilizing/destabilizing non-covalent interactions within the complexes, used as models for the junction nodes. It was shown that the presence of the amphiphilic chains enhanced the interaction behavior, increasing the binding constant more than 200-fold. Stability of the junction nodes was significantly affected by the host fit and guest orientation, host-guest interactions and desolvation effects. This combined approach can be adopted for obtaining a detailed understanding of the mechanisms governing soft associations in nanogel materials and other nanostructures such as targeted nanoparticles [124].

**Figure 7:**
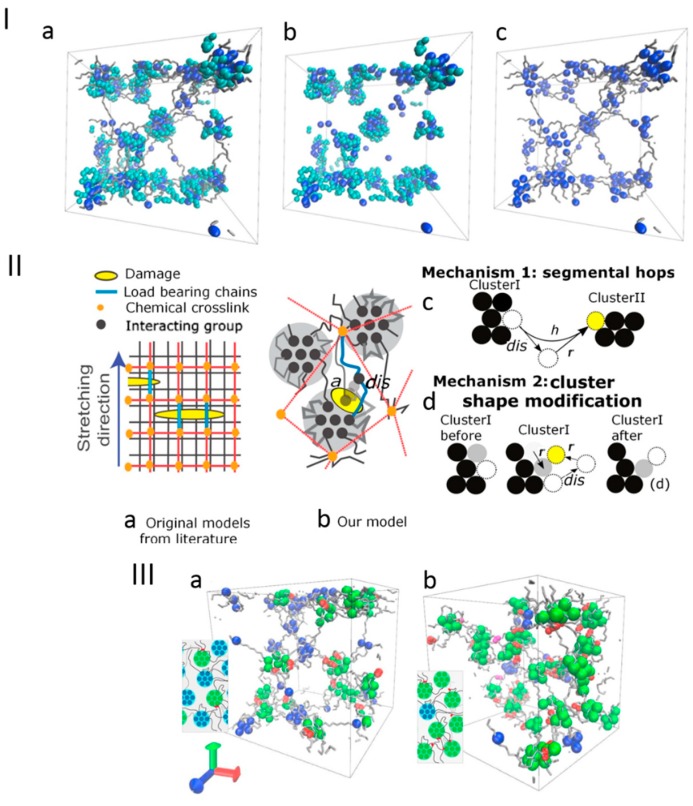
(**I**) Representative configurations of the crosslinked network at the principal structural parameter (cpc) of 0.00. Dark blue: strongly physically interacting groups, light blue: shells of clusters, gray: flexible chains. (**I**-**a**) dry phase of the simulation box, (**I**-**b**) the clusters, (**I**-**c**) the flexible chains. (**II**) Schematic representation of the models reported in (**II**-**a**) literature and by the authors (**II**-**b**), the latter describing physically/chemically crosslinked networks. Different recovery modes of the structure are also illustrated and include the (**II**-**c**) exchange of atomic groups between clusters, and the (**II**-**d**) reshaping of clusters by dissociation and association to the same cluster. (**III**) Snapshots of hybrid networks showing the chemical crosslinks (red) at *cpc* values of 0.7 (**III**-**a**) and 3.05 (**III**-**b**). The physically interacting groups without chemical crosslinks are represented in dark blue, while those with chemical crosslinks in their vicinity are in green. Retrieved from ref. [126] with permission from Elsevier.

Zidek et al. [126] have evaluated the structural deformation of hybrid crosslinked hydrogels, resorting to MD calculations. A mechanism of spontaneous response to damage allowing for the network structure recovery was proposed, based on the dissociation of interacting groups from physical clusters (see Figure 7). The mechanisms of structural recovery were identified as segmental hops and cluster shape modifications. In the former, a transition of atomic groups from one cluster to another was described, leading to fast stabilization of the structure, while the latter correspond to changes of the cluster shape, displaying higher resistance to damage.

MD and coarse-grained models have also been used to inspect the deformation ability of hydrogels loaded with magnetic nanoparticles in external magnetic fields, considering a set of different factors, such as the shape and arrangement of the magnetic particles in the gel, and the polymer network topology. It was concluded that, in contrast to uniaxial gels, in isotropic gels, clustering was reduced by an external magnetic field, as the interactions between particles were repulsive. In ellipsoidal gels, the uniaxial microstructure cancelled the expected deformation. With regard to the polymer network topology it was suggested that the microstructure was more stable as the polymers associated close and adjacent magnetic particles. When the external field was removed after crosslinking, the microstructure of the ferrogel preserved uniaxiality [127].

**Figure 8:**
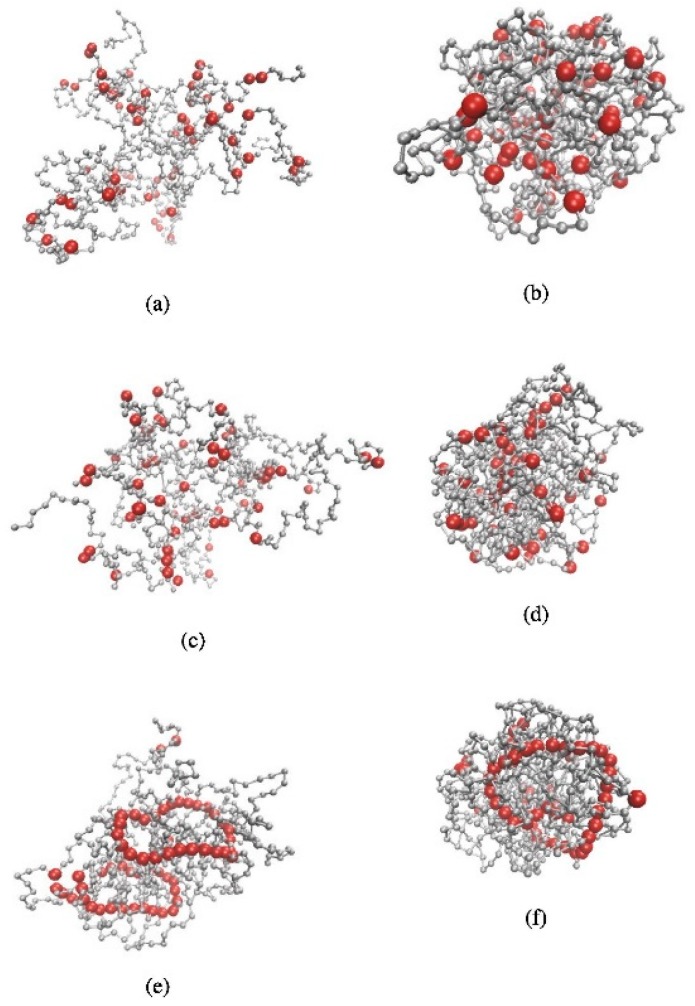
Representative microgel configurations obtained from the simulations of the microgels with magnetic particles (red), considering different degrees of crosslinking (N_cros_ = 20, panels (**a**,**c**,**e**), N_cros_ = 200, panels (**b**,**d**,**f**)) and different strengths of dipolar interactions λ (1–8). Retrieved from ref. [128] with permission from Elsevier.

In another study, Minina et al. [128] studied the effect of the interaction and aggregation between magnetic particles, on the structural and physical properties (e.g., microstructure, radius of gyration and magnetic susceptibility) of microgels (Figure 8). The latter were composed of varying concentrations of magnetic particles and displayed different degrees of crosslinking. It was shown that increasing the strength of dipolar interactions the size of microgels decreases. Deformation of microgels was also facilitated by the decreased of crosslinking. The magnetic response strongly decreased in highly crosslinking polymer networks. Such results allowed to understand important effects of combining magnetic end elastic properties in soft-matter.

## 5. Conclusions

In brain tumors, chemotherapy is still a limited treatment option, as lack of effectiveness and systemic toxicities usually stand out as main drawbacks. Tumor resection also proves to be insufficient, considering the high rates of recurrence. New therapy approaches are required, and hydrogels proved to be a potential weapon against brain tumors. Their unique characteristics, including high biocompatibility, biodegradability and response to stimuli, makes them excellent platforms for either localized and systemic drug delivery applications. On one hand, injectable macroscopic hydrogels containing nanostructured DDS show a localized and controlled delivery of drug to the tumor, reduced toxicity in healthy tissues and an effective inhibition of tumor growth and recurrences. On the other hand, to overcome the use of extremely invasive procedures, tailored nanogels became a very appealing strategy to efficiently deliver chemotherapeutic agents to neoplastic brain cells. Combining both hydrogel and nanoparticle characteristics, they have successfully demonstrated the ability of crossing the BBB and suffering preferential uptake, thus identifying and killing tumor cells, without compromising healthy tissues. Proof-of-concept demonstrations through in vivo models are encouraging, although more studies are required to pave a successful translation to clinical practice.

From in silico perspective, the design of advanced targeted nanomedicines based on polymer networks and nanostructures is still a challenging task, with a broad range of physical/chemical and therapeutic properties to be understood and controlled. As such, the insights gathered from simulation and modelling can be crucial to rationalize relevant interactions and dynamic behavior of these type of nanosystems, fostering the development of more effective formulations.

## Figures and Tables

**Table 1:** Hydrogel-based system as therapeutic or diagnostic strategies against brain tumors.

Delivery System	Hybrid System	Carried Agent	Route of Admin.	Trigger	Main Achievements	Ref.
**Hydrogel matrices containing nanostructured DDS**						
**Polymeric Micelles**	PEG_750_-p(CL-*co*-TMC) micelles + PEG-DMA-based in situ hydrogel	TMZ	Local Delivery	UV light	Fast in situ photopolymerization; sustained release of TMZ over 1 week; potent in vivo antitumor efficacy.	[28]
	mPEG-PDLLA micelles + macroporous gelatin hydrogel	PTX	Local Delivery	Enzymatic	Controlled release of PTX and high inhibition of tumor cell proliferation in glioma C6 cells.	[29]
	HA NPs + collagen hydrogel	Lentivirus	Local Delivery	-	HA within the hydrogel increased the lentivirus activity for 72 h and delayed its release; Transduction activity in invasive C6 glioma cells with the hydrogel was ~80% of the control.	[30]
	BSA NPs + CMC-*g*-PNIPAAmMA and DTPAGd/bPEI hydrogel	PTX + EPI	Local Delivery	Temp.	MBR 614 cell line: sustained drug release and inhibition of tumor cells growth; human glioma U87 MG tumor-bearing mouse: effective tumor reduction and average survival increase.	[31]
	Silica NPs + PEG-based hydrogel	CPT	Local Delivery	UV light	U87 MG cells: marked decreased in cell viability.	[32]
	mPEG-PLGA NPs + PF127 hydrogel	PTX + TMZ	Local Delivery	Temp.	Drug release was controlled by gel composition; High growth inhibition and apoptosis-inducing effects in both U87 MG and C6 cell lines.	[33]
**Magnetic NPs**	Fe_3_O_4_ NPs + PEG-based hydrogel	PTX	Local Delivery	AMF	Effective delivery of PTX simultaneously with hyperthermia in M059K glioma cells.	[34,35]
	P-CoFe_2_O_4_ NPs + PPZ hydrogel	SN-38	Local Delivery	-	Drug sustained release; long-term inhibition of tumor growth in U87 MG tumor-bearing mice; proved MR imaging abilities.	[36,37]
**Liposomes**	Liposomes + P(NIPAAm-*co*-BMA) and PEG-based hydrogel	DOX	Local Delivery	Temp.	Increased sustained release over 52 days; Significant reduction of tumor growth (38 days vs. 12 days with free DOX in hydrogel).	[27]
**LNC**	Lipid nanocapsule-based hydrogel	GemC_12_	Local Delivery	-	Sustained drug release over one month with a significant increase of survival/reduction of recurrences in tumor xenograft models.	[38,39]
**SLNs**	Anti-EGFR-SLNs + in situ gel	CP	Intranasal	-	The optimized formulation presented good viscosity, gelling strength, drug content as well as favorable adhering properties to the nasal mucosa, stability and dissolution profile, among others.	[40]
**Hydrogel Nanoparticles**						
**Hydrogel Nanoparticles**	Polyglycerol-scaffold nano-polyplexes in polymeric nanogels	miR-34a	Local Delivery	pH/redox	Significant reduction in tumor size growth of abdominal GBM xenograft models.	[41]
	Liposome-templated hydrogel NPs	CRISPR/Cas9	IV	-	Controlled release of DNA and protein; Marked cytotoxicity over U87 MG cells; reduction of tumor growth and improvement of overall survival of mice models.	[42]
	PEI-modified PAA-based hydrogel NPs	CIS	-	-	Uptake of nanogels promoted by PEI-9L glioma cells interaction; higher toxicity of NPs over glioma cells than free CIS.	[43]
	Fe_3_O_4_ NPs loaded MPNA nanogel	Cy5.5	IV	pH/Temp.	Proved MR/fluorescence imaging abilities; Good and specific uptake by C6 glioma cells in rat model; cellular uptake favored at pH 6.8 (tumor environment) using lactoferrin.	[44]
	PAA-based hydrogel NPs	CB	IV	-	Covalently linked CB nanoparticles, functionalized with F3 peptide and PEG, effectively target and clearly identify GBM cells.	[45]
	F3 peptide-conjugated co(CEA-AAm) nanogel	DOX	-	-	Surface modification with F3 peptide increased NPs uptake by 9 L glioma cells; NPs show controlled release of DOX (42% in the first 24 h).	[46]
	Anti-Cx43 and anti-BSAT1-conjugated nanogels	CIS	IV	-	Sustained release of CIS (50% after one week); Despite the lower cytotoxicity over C6 cells than free CIS, NPs improved the overall survival of 101/8 cells implanted in rats.	[47]
	cRGD-decorated PVA nanogels	DOX	IV	pH/redox	Triggered release of DOX caused by low pH and redox environment; cRGD modified nanogels effectively reduce tumor growth in vivo.	[48]
	Alginate nanogel co-loaded with gold nanoparticles	CIS	-	X-Ray	Higher toxicity on U87 MG cells, when compared to free CIS; marked apoptotic effect after X-ray irradiation.	[49]
	Fe^3+^-crosslinked pentaerythritol poly-(caprolactone)-*b*-poly(acrylic acid) nanogels	DOX	IV	Light	Nanoparticles effectively release DOX after the exposure of the tumor to light, with 91% of tumor growth inhibition and no adverse effects.	[50]
**Hydrogel matrices containing microstructured DDS**						
**Microspheres**	PLGA microspheres + TGP hydrogel	CPT	Local Delivery	Temp.	Sustained drug release of CPT at the tumor site; hydrogel administration and tumor resection significantly increased overall survival of the models (over 60 days).	[24,25]
	PLGA microspheres + alginate hydrogel	PTX	Local Delivery	-	After a low initial burst, microspheres exhibited a controlled drug release over more than 60 days; higher cytotoxicity over C6 cells when compared to reference; reduction of tumor volume in tumor-bearing models.	[26]
	PLGA microspheres + P(NIPAAm-*co*-BMA) and PEG-based hydrogel	DOX	Local Delivery	Temp.	Increased sustained release over 30 days; significant reduction of tumor growth (32 days vs. 12 days with free DOX in hydrogel).	[27]

Key: PEG_750_-p(CL-*co*-TMC) = poly(ethylene glycol) 750-(poly(ε-caprolactone-*co*-trimethylene carbonate)); PEG = poly(ethylene glycol); DMA = dimethacrylate; TMZ = temozolomide; UV = ultraviolet; mPEG = methoxypoly(ethylene glycol); PDLLA = poly(d,l-lactide); PTX = paclitaxel; C6 cells = rat glioma cells; HA = hydroxylapatite; NPs = nanoparticles; BSA = bovine serum albumin; CMC-g-PNIPAAmMA = carboxymethyl cellulose (CMC)-grafted poly(*N*-isopropylacrylamideco-methacrylic acid); DTPAGd/bPEI = gadopentetic acid/branched polyethylenimine; EPI = epirubicin; Temp. = temperature; MBR 614 cell line = human brain tumor cells; U87 MG = human likely glioblastoma cells; CPT = camptothecin; PLGA = poly(lactic-*co*-glycolic acid); PF127 = Pluronic^®^ F127; AMF = alternating magnetic field; M059K glioma cells = human brain malignant glioblastoma cells; P-CoFe_2_O_4_ = PEGylated cobalt ferrite; PPZ = poly(organophosphazene); SN-38 = 7-ethyl-10-hydroxycamptothecin; MR = magnetic resonance; P(NIPAAm-*co*-BMA) = poly(*N*-isopropylamide-*co*-*n*-butylmethacrylate); DOX = doxorubicin; LNC = lipid nanocapsule; GemC_12_ = lauroyl-gemcitabine (prodrug); SLNs = solid lipid nanoparticles; EGFR = epidermal growth factor receptor; CP = Cyclophosphamide; miR-34a = microRNA encoded by the human MIR34A gene and recognized as a regulator of tumor suppression; GBM = glioblastoma; CRISPR/Cas9 = Clustered Regularly Interspaced Short Palindromic Repeats/CRISPR associated protein 9 system; IV = intravenous; DNA = deoxyribonucleic acid; PEI = polyethylenimine; PAA = polyacrylamide; CIS = cisplatin; 9 L glioma cells = rat gliosarcoma cells; MPNA = poly(*N*-isopropylacrylamide-*co*-acrylic acid; Cy5.5 = Cyanine5.5 NHS; CB = Coomassie Brilliant Blue G-250; F3 peptide = 31 amino acid peptide with tumor homing capability; co(CEA-AAm) = 2-carboxyethyl acrylate-acrylamide copolymer; Cx43 = Gap junctional protein Connexin 43; BSTAT1 = brain-specific anion transporter; 101/8 cells = rat glioblastoma cells; cRGD = cyclo(Arg-Gly-Asp) peptide; PVA = poly(vinyl alcohol); PLGA = poly(d,l-lactide-*co*-glycolide); TGP = thermoreversible gelation polymer.

**Table 2:** Overall features assigned to hydrogel nanoparticles with impact on drug delivery [80,84].

Critical Quality Attributes	Justification
Easy to synthetize	They can be easily scaled up for large-scale production, in addition to being an eco-friendly chemistry approach.
Nanosize	Their small size eases the passage through biological barriers and avoids clearance by phagocytic cells, thus increasing their blood circulation time.
Viscoelasticity	Given their ability to deform, i.e., to switch between solid-like and liquid-like states, hydrogel nanoparticles pass more easily through biological barriers and cell membranes.
Swelling capacity	Occurring in aqueous fluids, swelling is controlled by chemical structure of the gel matrix and its crosslinking degree, as well as environmental variables (temperature, pH and ionic strength).
Response to stimuli	They can respond to biological stimuli, ensuring site-specific and controlled release of drugs. Such response involves changes in physicochemical properties of the hydrogel nanoparticles (swelling, permeability, viscoelasticity).
Encapsulation stability	Their crosslinked structure allows an extend drug circulation time in bloodstream, protecting drugs from enzymatic/chemical degradation.
Passive and active targeting	A wide range of bioactives (drugs, peptides, proteins, antibodies and vaccines) can be coupled to the surface of hydrogel nanoparticles in order to target specific tissues. In addition, stimuli-responsive hydrogels (as referred above) are another strategy of active targeting. Extravasation in the pathological sites and retention in the microvasculature could represent passive targeting approaches of hydrogel nanoparticles. All of them increase therapeutic efficacy and reduce undesired effects.
**Controlled and sustained drug release**	Internal crosslinking modulation of hydrogel nanoparticle networks can control both drug loading capacity and drug release.
Minimal toxicity	They are biocompatible, non-immunogenic and biodegradable.
